# Proteomic analysis of bioprinted pancreatic tumor models under different nutrient conditions

**DOI:** 10.3389/fbioe.2026.1921652

**Published:** 2026-10-05

**Authors:** Busra Buyuk, Ming Li, Xueer Song, Kaiming Ye

**Affiliations:** 1 Department of Biomedical Engineering, Watson College of Engineering and Applied Science, Center of Biomanufacturing for Regenerative Medicine, Binghamton University, State University of New York (SUNY), Binghamton, NY, United States; 2 Department of Bioengineering, Recep Tayyip Erdoğan University, Rize, Türkiye

**Keywords:** 3D bioprinting, cancer-associated fibroblasts, metabolic adaptation, nutrient stress, pancreatic ductal adenocarcinoma (PDAC), proteomics, tumor microenvironment

## Abstract

**Background:**

Pancreatic ductal adenocarcinoma (PDAC) is characterized by a dense stromal microenvironment that restricts nutrient availability and contributes to tumor adaptation. Cancer-associated fibroblasts (CAFs), extracellular matrix components, and tumor cells participate in reciprocal interactions that influence the metabolic state of PDAC tumors.

**Objective:**

This study investigated nutrient-associated proteomic changes in three-dimensional (3D) bioprinted PDAC–CAF tumor constructs and examined alterations in metabolic and cell-junction-related pathways under nutrient-avid and nutrient-deprived conditions.

**Methods:**

Three-dimensional bioprinted pancreatic tumor constructs composed primarily of PANC1 cells and cancer-associated fibroblasts (CAFs) were cultured under nutrient-avid (N) and nutrient-deprived (N−) conditions. Constructs were monitored at Days 0, 6, and 12, and Day 6 was selected for comparative proteomic analysis as an intermediate stage at which nutrient-associated structural divergence was evident while both tumor and stromal compartments remained detectable. Label-free high-resolution LC–MS/MS proteomic profiling was followed by KEGG pathway enrichment and complementary bioinformatic analyses. Selected junction-associated findings were further examined by quantitative real-time PCR.

**Results:**

Distinct nutrient-associated proteomic signatures were observed in the bioprinted tumor constructs. Nutrient-avid conditions were associated with enrichment of pathways including pantothenate and CoA biosynthesis, β-alanine metabolism, and arginine and proline metabolism. Nutrient-deprived conditions showed enrichment of pathways related to unsaturated fatty-acid biosynthesis, fatty-acid elongation, riboflavin metabolism, folate-associated metabolism, and terpenoid backbone biosynthesis. Nutrient-associated differences were also observed among proteins assigned to gap-junction, adherens-junction, focal-adhesion, tight-junction, and central-carbon-metabolism pathways. Selected junction-associated proteomic observations were supported at the transcript level by qPCR, although concordance between mRNA and protein abundance varied among targets.

**Conclusion:**

Nutrient availability was associated with distinct metabolic and junction-related proteomic signatures in 3D bioprinted PDAC–CAF constructs. These findings provide hypothesis-generating evidence of nutrient-dependent molecular remodeling within a multicellular PDAC model. Functional perturbation studies will be required to determine the causal and therapeutic relevance of the candidate proteins and pathways identified here.

## 1 Introduction

Pancreatic ductal adenocarcinoma (PDAC) is the most common form of pancreatic cancer and remains associated with poor clinical outcomes despite advances in diagnosis and therapy. A defining feature of PDAC is its extensive desmoplastic tumor microenvironment (TME), which contains abundant extracellular matrix (ECM), fibroblastic stromal populations, immune cells, vascular components, and other non-malignant cell types. This highly structured microenvironment influences nutrient and oxygen availability, mechanical properties, intercellular signaling, and therapeutic response. Consequently, PDAC biology cannot be fully understood by considering malignant epithelial cells independently of their surrounding stromal context (Sarantis et al., 2020; Sherman and Beatty, 2023; Truong and Pauklin, 2021).

Cancer-associated fibroblasts (CAFs) constitute a major cellular component of the PDAC stroma; however, CAFs do not represent a single uniform cell population. Original studies have demonstrated the coexistence of functionally distinct fibroblast states within PDAC. Öhlund et al. identified spatially and functionally distinct myofibroblastic CAFs (myCAFs) and inflammatory CAFs (iCAFs), while subsequent single-cell analyses identified an antigen-presenting CAF (apCAF) population expressing MHC class II-related genes (Öhlund et al., 2017; Elyada et al., 2019). Human PDAC-derived CAFs also exhibit substantial inter- and intra-tumoral heterogeneity, indicating that multiple stromal programs may coexist within individual tumors (Neuzillet et al., 2019). These populations can differ in localization, signaling programs, ECM production, inflammatory activity, and interactions with malignant cells. Thus, apparently conflicting effects attributed to CAFs may partly reflect fibroblast heterogeneity as well as differences in tumor stage, spatial organization, and experimental model. The present bioprinted model captures selected tumor–fibroblast interactions but does not reproduce the complete cellular heterogeneity of the human PDAC microenvironment.

One consequence of PDAC desmoplasia and abnormal vascular organization is the development of spatially heterogeneous nutrient conditions. Tumor regions with limited perfusion may experience reduced availability of glucose, amino acids, oxygen, and other metabolic substrates. PDAC cells can nevertheless survive within these metabolically restrictive environments by altering nutrient acquisition and utilization. Stromal cells may further modify this response by releasing metabolites, extracellular-matrix components, and signaling factors that can be utilized by neighboring tumor cells. Stromal cues have been shown to alter the PDAC epigenome and metabolome, while CAF-derived metabolites and lipids can support tumor-cell survival and growth under nutrient-limited conditions (Sherman et al., 2017; Francescone et al., 2021; Auciello et al., 2019; Han et al., 2024)**.** Accordingly, metabolic adaptation in PDAC is shaped not only by cancer-cell-intrinsic programs but also by bidirectional interactions between malignant and stromal compartments.

PDAC cells exhibit substantial metabolic plasticity. Oncogenic and microenvironmental signals can alter glucose utilization, amino-acid metabolism, mitochondrial function, redox regulation, and lipid metabolism (Pavlova et al., 2022; Vander Heiden et al., 2009). Importantly, metabolic states in PDAC are heterogeneous rather than uniform. Broad metabolomic profiling has identified distinct PDAC metabolic subtypes, including relatively glycolytic and lipogenic states with differences in glucose and glutamine utilization, mitochondrial function, and lipid synthesis (Daemen et al., 2015)**.** Additional studies have linked glycolysis–cholesterol synthesis programs and tumor/microenvironment characteristics to PDAC biological behavior and clinical outcome (Karasinska et al., 2020; Puleo et al., 2018). Nutrient limitation may therefore alter the relative representation or utilization of metabolic pathways without necessarily producing a single universal metabolic phenotype.

Lipid metabolism is particularly relevant to cellular adaptation under metabolic stress because fatty acids and complex lipids contribute to membrane synthesis, energy storage, redox regulation, and intracellular signaling. Activated pancreatic stromal cells undergo extensive lipid remodeling and can release lysophospholipids that support PDAC growth and signaling (Auciello et al., 2019). More recent work has further demonstrated that CAF-derived unsaturated lipids can support PDAC membrane homeostasis and survival under nutrient and oxygen limitation (Han et al., 2024). However, changes in pathway-associated protein abundance do not directly establish metabolic flux. Therefore, proteomic enrichment of lipid-associated pathways should be interpreted as evidence of altered molecular representation of these pathways rather than direct demonstration of increased lipid synthesis or oxidation.

In addition to metabolic regulation, nutrient availability may influence cell–cell and cell–matrix organization. Adherens junctions and tight junctions contribute to epithelial organization and cell polarity, whereas focal adhesions mediate interactions between cells and the extracellular matrix. Gap-junction-associated signaling can contribute to intercellular communication. Alterations in proteins assigned to these pathways may therefore accompany structural remodeling of tumor–stromal constructs during nutrient stress. Nevertheless, pathway association alone does not establish changes in junctional function, and functional validation is required to determine the biological consequences of individual protein alterations.

To investigate nutrient-associated molecular responses in a spatially organized tumor–stromal environment, we employed the previously established three-dimensional bioprinted “sea-and-island” pancreatic tumor construct (sandi PTC) (Li et al., 2024). In this model, PDAC cell islands are embedded within and surrounded by a dense CAF/ECM-containing stromal compartment designed to reproduce selected structural and nutrient-diffusion characteristics of the desmoplastic PDAC microenvironment (Li et al., 2024). The platform was previously characterized using PANC-1 and CAPAN-2 cells, representing poorly differentiated/basal-like and well-differentiated/classical PDAC models, respectively (Li et al., 2024).

Previous characterization of the sandi PTC further demonstrated nutrient-dependent changes in CAF-associated phenotypes. In particular, α-SMA-high/IL-6-low CAF-associated signatures were enriched under nutrient-avid conditions, whereas α-SMA-low/IL-6-high patterns consistent with an inflammatory-like CAF state were observed under nutrient-deprived conditions (Li et al., 2024). These earlier findings establish the biological context of the model used in the present proteomic study; however, comprehensive CAF subtype profiling was not independently repeated in the current analysis.

The sandi PTC platform was also previously shown to restrict free molecular diffusion through its CAF/ECM-rich compartment, undergo nutrient-dependent ECM remodeling, and exhibit distinct proliferative and apoptotic responses as assessed using Ki67 and cleaved caspase-3 staining (Li et al., 2024). These prior characterization data provided the experimental basis for using the model to investigate nutrient-associated proteomic changes in the present study.

## 2 Methods

### 2.1 Cell culture and nutrient modulation

Human PDAC cell lines PANC-1 and CAPAN-2 were used in the previously established sea-and-island pancreatic tumor construct as described by Li et al. (Li et al., 2024). PANC-1 and CAPAN-2 represent PDAC models with relatively poorly differentiated/basal-like and well-differentiated/classical characteristics, respectively.

The human PDAC-derived CAF population used in the sandi PTC platform originated from an untreated patient’s surgical specimen and was obtained through Fox Chase Cancer Center. The parental CAF population and related derivatives have previously been extensively characterized by the originating investigators, including SNP-based authentication. Cells were maintained according to the previously established culture procedures and were routinely used within 10 passages following recovery from cryostorage [16 and references therein].

Prior characterization of these CAFs within the sandi PTC demonstrated nutrient-associated differences in α-SMA and IL-6 expression consistent with dynamic myofibroblastic-like and inflammatory-like CAF states (Li et al., 2024). The present study did not independently perform comprehensive molecular CAF subtype classification and therefore does not assign the observed proteomic alterations to a specific CAF subtype.

The nutrient-avid condition consisted of DMEM containing 4.5 g/L glucose, 4 mM L-glutamine, 10% FBS, and penicillin–streptomycin. The nutrient-deprived condition contained 2 g/L glucose and lacked supplemental glutamine and FBS, as described for the experimental model.

### 2.2 Fabrication of sea-and-island pancreatic tumor constructs

Three-dimensional sea-and-island pancreatic tumor constructs (sandi PTCs) were generated using an extrusion-based bioprinting approach. A composite bioink was prepared by combining type I rat-tail collagen and gelatin methacryloyl (GelMA). Final concentrations within the printable hydrogel were adjusted to approximately 2 mg/mL collagen and 6 mg/mL GelMA. Lithium phenyl-2,4,6-trimethylbenzoylphosphinate (LAP) was incorporated as a photoinitiator to enable UV-mediated cross-linking. CAFs were suspended within the stromal bioink phase at a final density of approximately 1.2 × 10^7^ cells/mL. Tumor “island” compartments were established by incorporating PDAC cells (PANC1 or CAPAN-2) into designated bioink regions at a density of approximately 6 × 10^7^ cells/mL. Bioprinting was performed using an air-extrusion 3D bioprinter equipped with a 22-gauge nozzle. Printing pressure was optimized to achieve continuous filament deposition. Following printing, constructs were exposed to 365-nm ultraviolet light for 2 min to induce photo crosslinking and structural stabilization. Printed constructs were transferred to ultra-low-attachment culture plates containing CAF maintenance medium and incubated under standard culture conditions. Constructs were initially maintained in nutrient-replete medium for 48 h to allow cellular adaptation and matrix consolidation. Subsequently, constructs were assigned to nutrient-avid or nutrient-deprived experimental groups and cultured without further medium replacement during the remaining experimental period.

### 2.3 Proteomic sample preparation and LC–ms/MS analysis

Peptide mixtures were analyzed by data-independent acquisition using parallel accumulation–serial fragmentation (dia-PASEF) on a Bruker timsTOF mass spectrometer coupled to a 60-min liquid chromatography gradient. Raw mass spectrometry data were processed using Spectronaut (Biognosys). Protein-group identifications were filtered at a 1% false discovery rate (FDR; Q-value ≤0.01). Label-free protein quantification was performed using the quantitative abundance values generated during data processing. A protein was considered reliably detected and retained for downstream analysis only when it was quantified in at least two of three biological replicates within a condition. The resulting protein-abundance dataset was used for differential protein-abundance and downstream pathway analyses.

### 2.4 Bioinformatics and pathway enrichment analysis

Differentially abundant proteins identified in each nutrient-avid versus nutrient-deprived pairwise comparison were subjected to Gene Ontology (GO) and Kyoto Encyclopedia of Genes and Genomes (KEGG) pathway enrichment analysis. Pathway enrichment scores were expressed as −log10 (p-value), and pathways with p < 0.05 were considered significantly enriched. For each significantly enriched pathway, proteins detected within the pathway showing condition-associated differences in abundance were mapped onto the corresponding KEGG pathway schematics (Figure 1-7). Original KEGG pathway maps are provided in the Supplementary Figures. Node coloring was used to distinguish proteins associated with the nutrient-avid and nutrient-deprived conditions.

**FIGURE 1:**
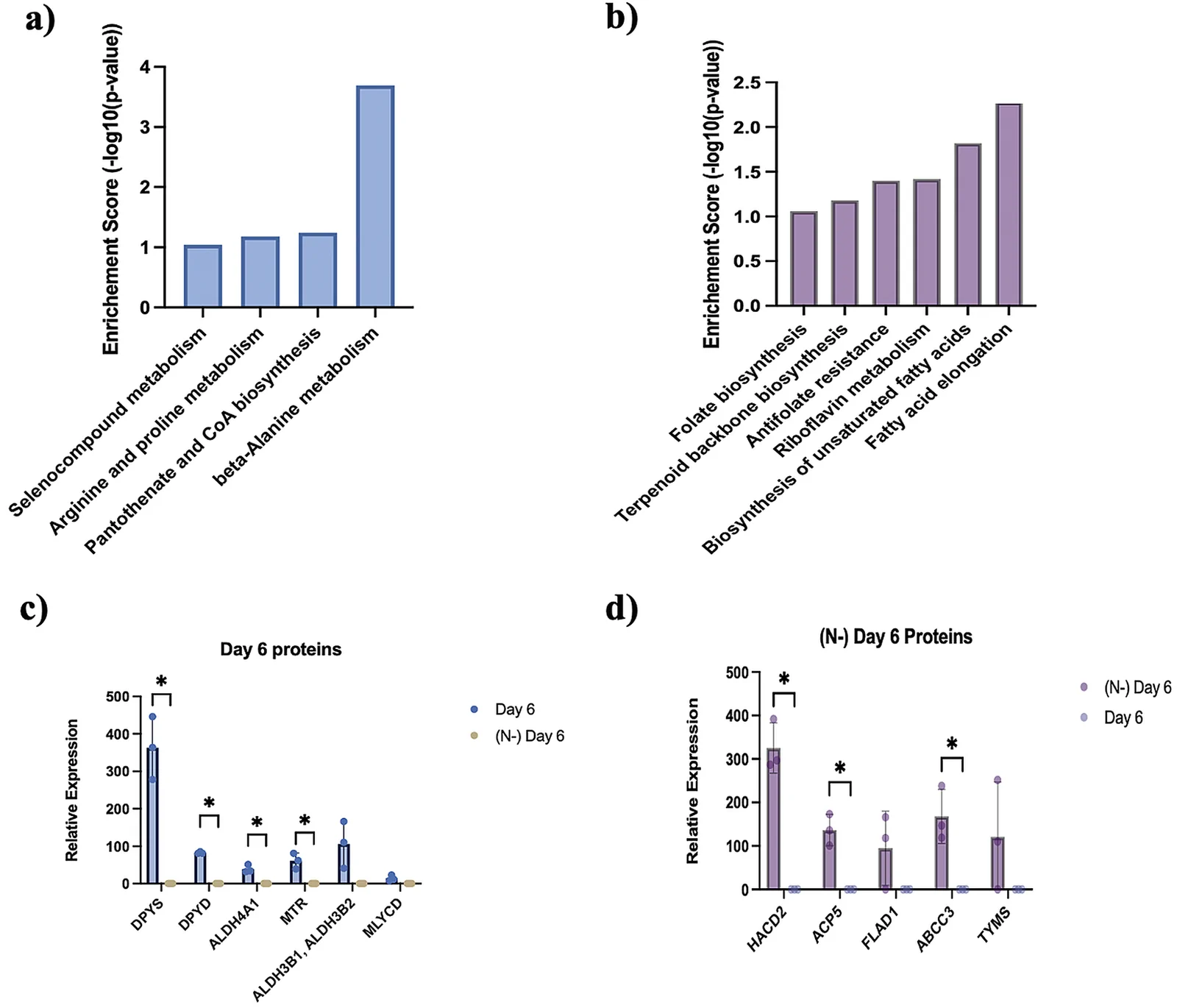
PANC1&CAF and enrichened metabolic signaling pathways on Day 6 under nutrient-avid or -deprived conditions. **(A)** The enriched metabolic signaling pathways identified in PANC1&CAF under nutrient-avid **(B)** or -deprived conditions. **(C)** The enriched metabolic signaling pathways’ exclusive protein expressions **(D)** or -deprived conditions. The bars show enrichment scores calculated by [−log10 (*p*-value)] for individual pathways. N = 3 for each group. p-value<0.05 is considered significantly enriched metabolic pathways. P < 0.05 (*), p < 0.01 (**), p < 0.001 (***).

**FIGURE 2:**
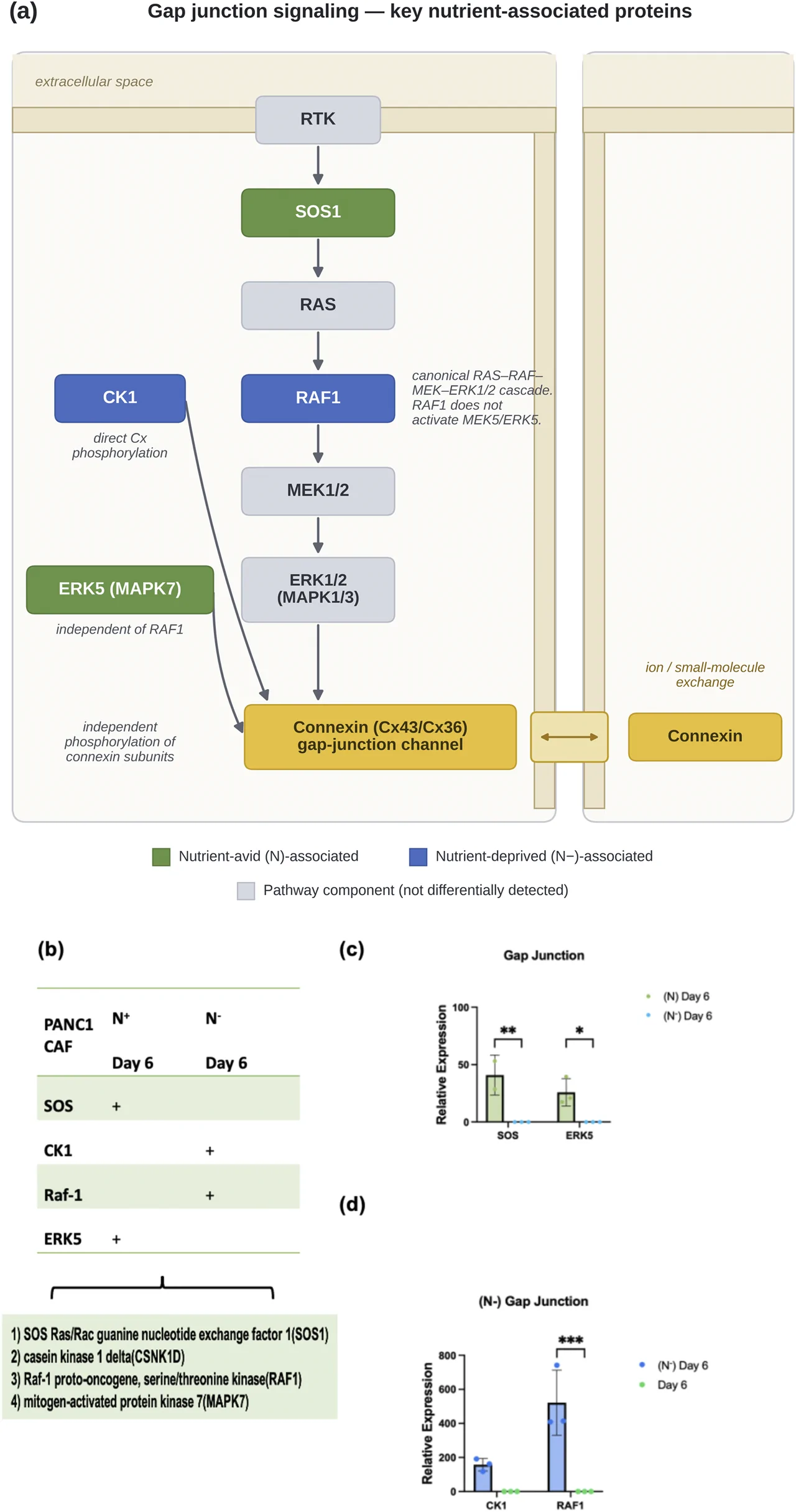
Alterations in gap junction-related proteins in response to nutrient availability. **(A)** Simplified schematic highlighting the principal nutrient-associated proteins identified in the proteomic analysis. Green indicates nutrient-avid (N)-associated proteins, blue indicates nutrient-deprived (N−)-associated proteins, and gray indicates pathway components included for context but not differentially detected. Original KEGG pathway maps are provided in Supplementary Figure S4 and S5. **(B)** Key nutrient-associated proteins detected at Day 6 **(C–D)** Relative protein abundance normalized to total protein concentration. Proteins detected in at least two of three biological replicates were considered reliably detected. Data are mean ± SD (n = 3). Statistical significance was determined by two-way ANOVA with Sidak’s multiple-comparisons test; p < 0.05, p < 0.01, p < 0.001.

**FIGURE 3:**
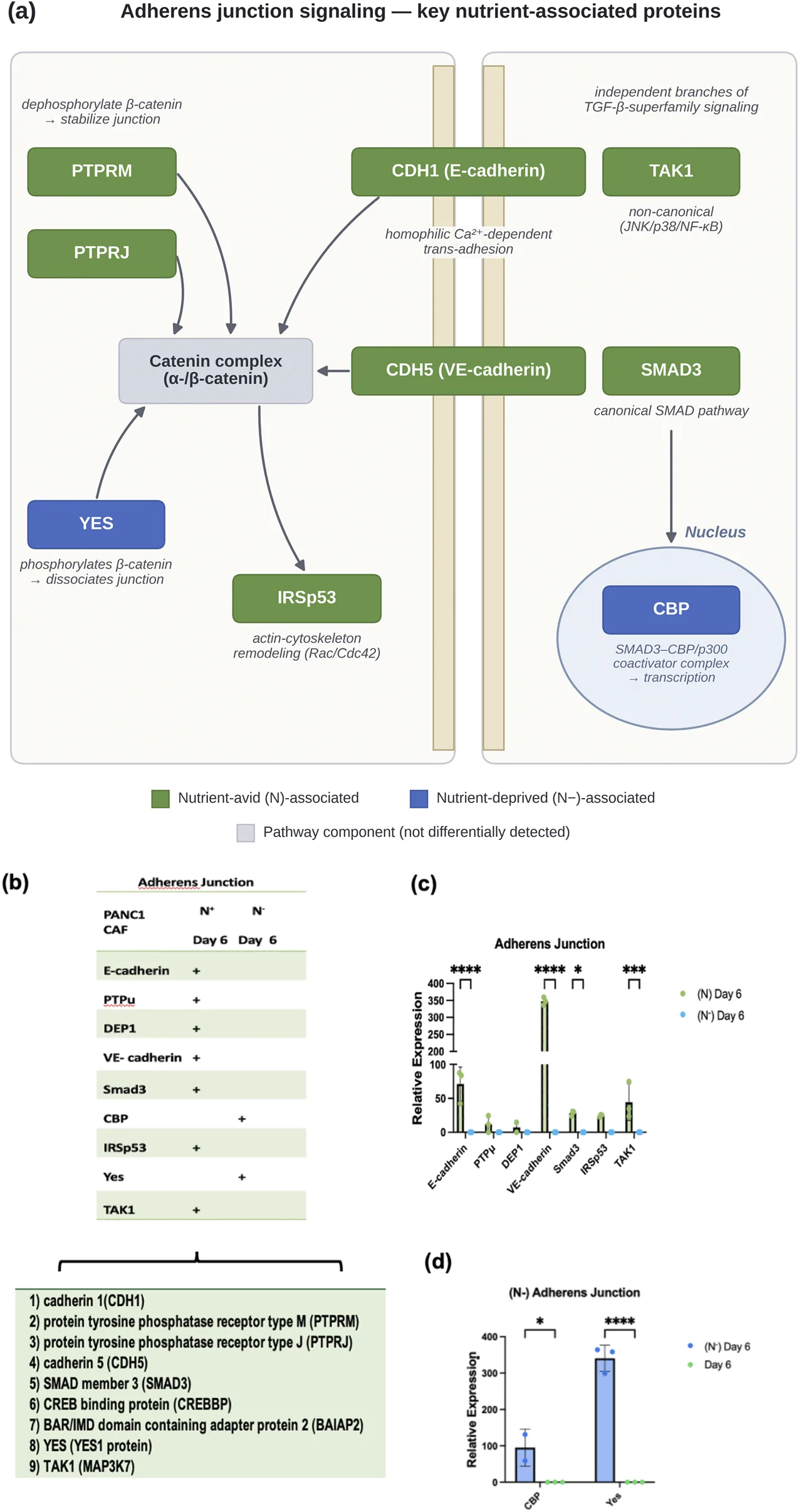
Alterations in adherens junction-related proteins in response to nutrient availability. **(A)** Simplified schematic highlighting the principal nutrient-associated proteins identified in the proteomic analysis. Green indicates nutrient-avid (N)-associated proteins, blue indicates nutrient-deprived (N−)-associated proteins, and gray indicates pathway components included for context but not differentially detected. Original KEGG pathway maps are provided in Supplementary Figure S2 and S3. **(B)** Key nutrient-associated proteins detected at Day 6 **(C–D)** Relative protein abundance normalized to total protein concentration. Proteins detected in at least two of three biological replicates were considered reliably detected. Data are mean ± SD (n = 3). Statistical significance was determined by two-way ANOVA with Sidak’s multiple-comparisons test; p < 0.05, p < 0.01, p < 0.001.

**FIGURE 4:**
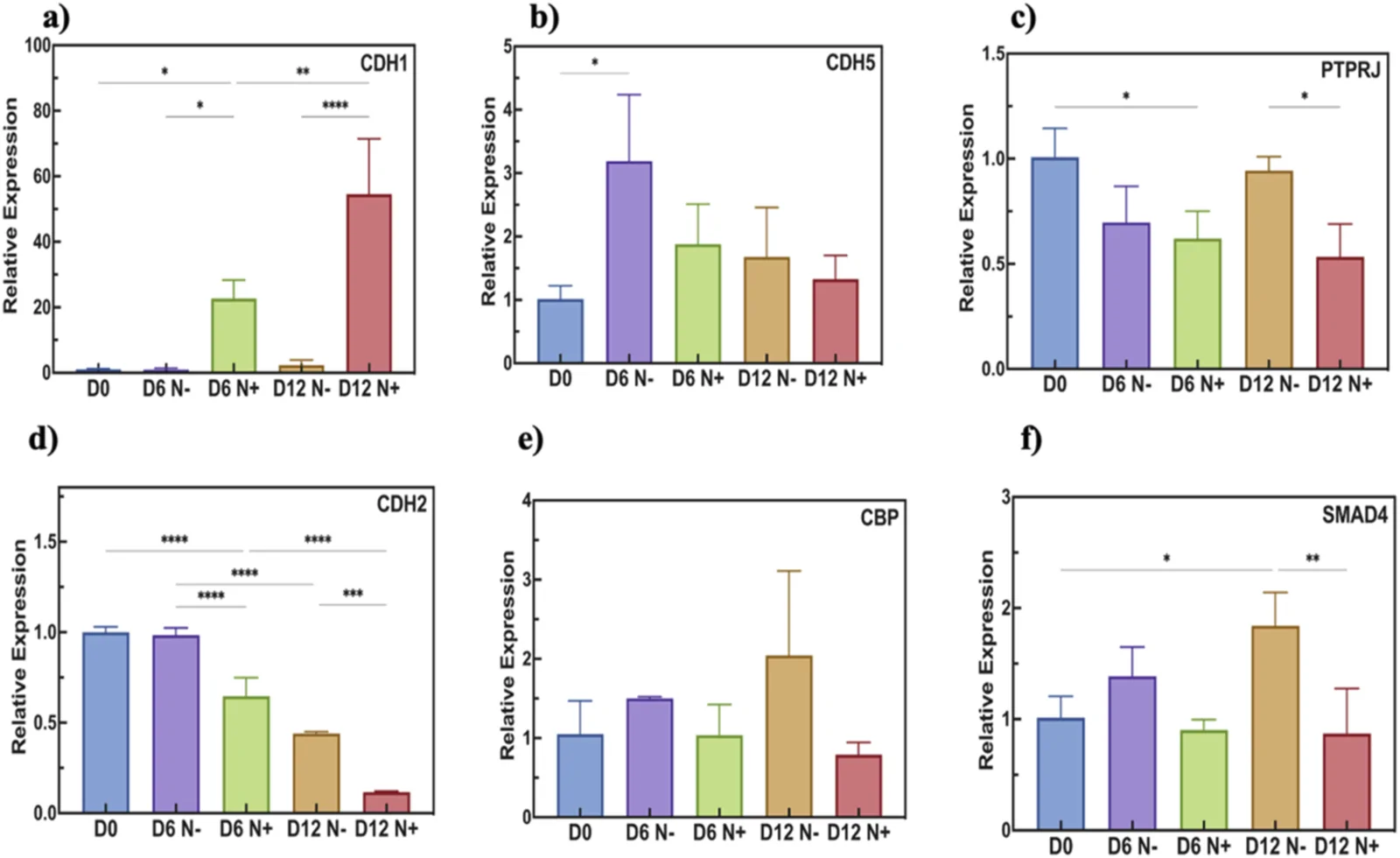
qPCR assessment of selected adherens junction–related genes. Relative mRNA expression of CDH1, CDH2, CDH5, PTPRJ, CBP, and SMAD4 in bioprinted PDAC–CAF constructs cultured under nutrient-avid (N) and nutrient-deprived (N−) conditions at Days 6 and 12. Gene expression was normalized to GAPDH and calculated using the ΔΔCt method. Data represent three independent biological replicates.

**FIGURE 5:**
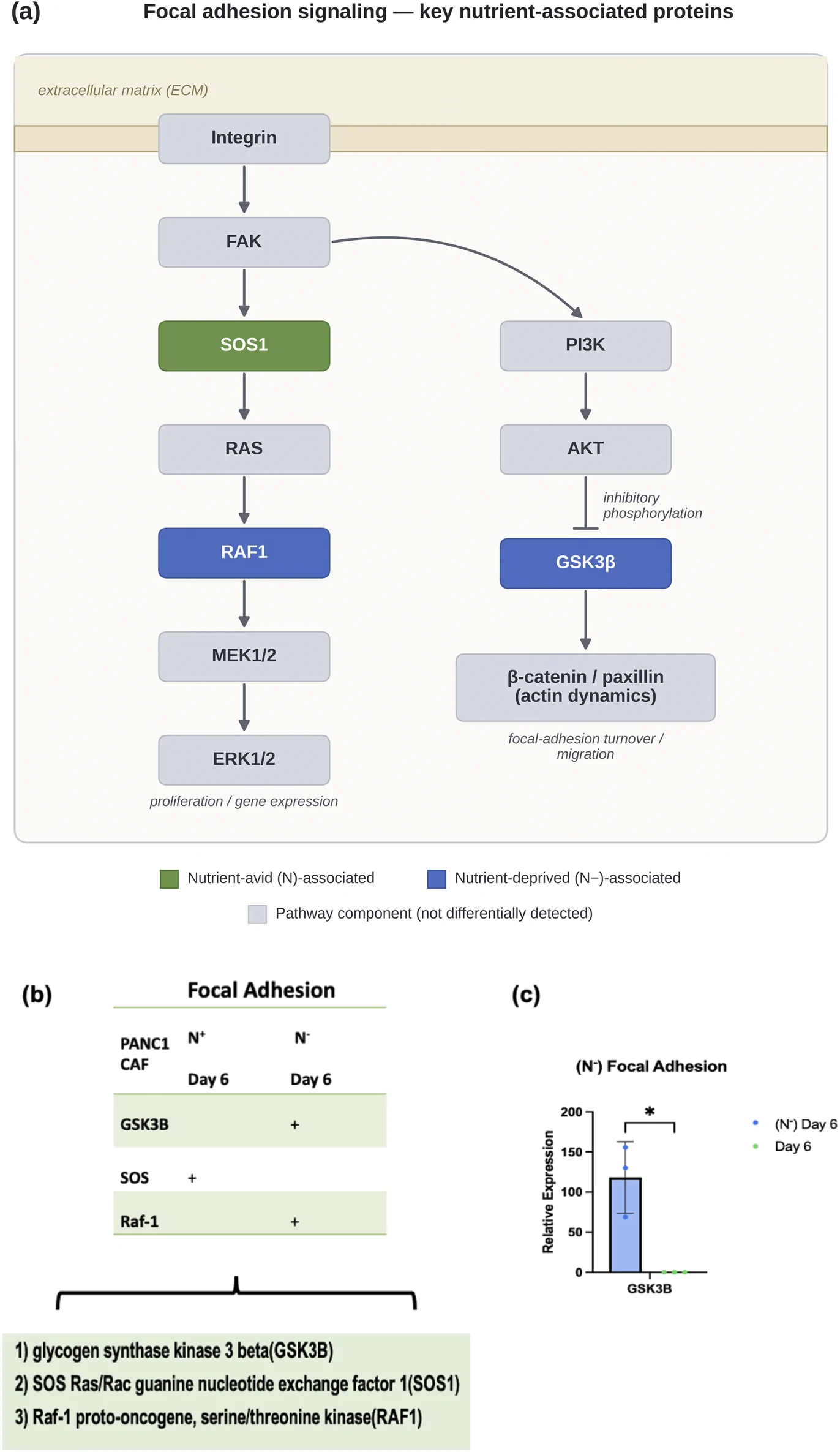
Alterations in focal adhesion-related proteins in response to nutrient availability. **(A)** Simplified schematic highlighting the principal nutrient-associated proteins identified in the proteomic analysis. Green indicates nutrient-avid (N)-associated proteins, blue indicates nutrient-deprived (N−)-associated proteins, and gray indicates pathway components included for context but not differentially detected. Original KEGG pathway maps are provided in Supplementary Figure S8 and S9. **(B)** Key nutrient-associated proteins detected at Day 6. **(C)** Relative protein abundance normalized to total protein concentration. Proteins detected in at least two of three biological replicates were considered reliably detected. Data are mean ± SD (n = 3). Statistical significance was determined using Welch’s *t*-test; *p* < 0.05, **p <** 0.01, ***p* <** 0.001.

**FIGURE 6:**
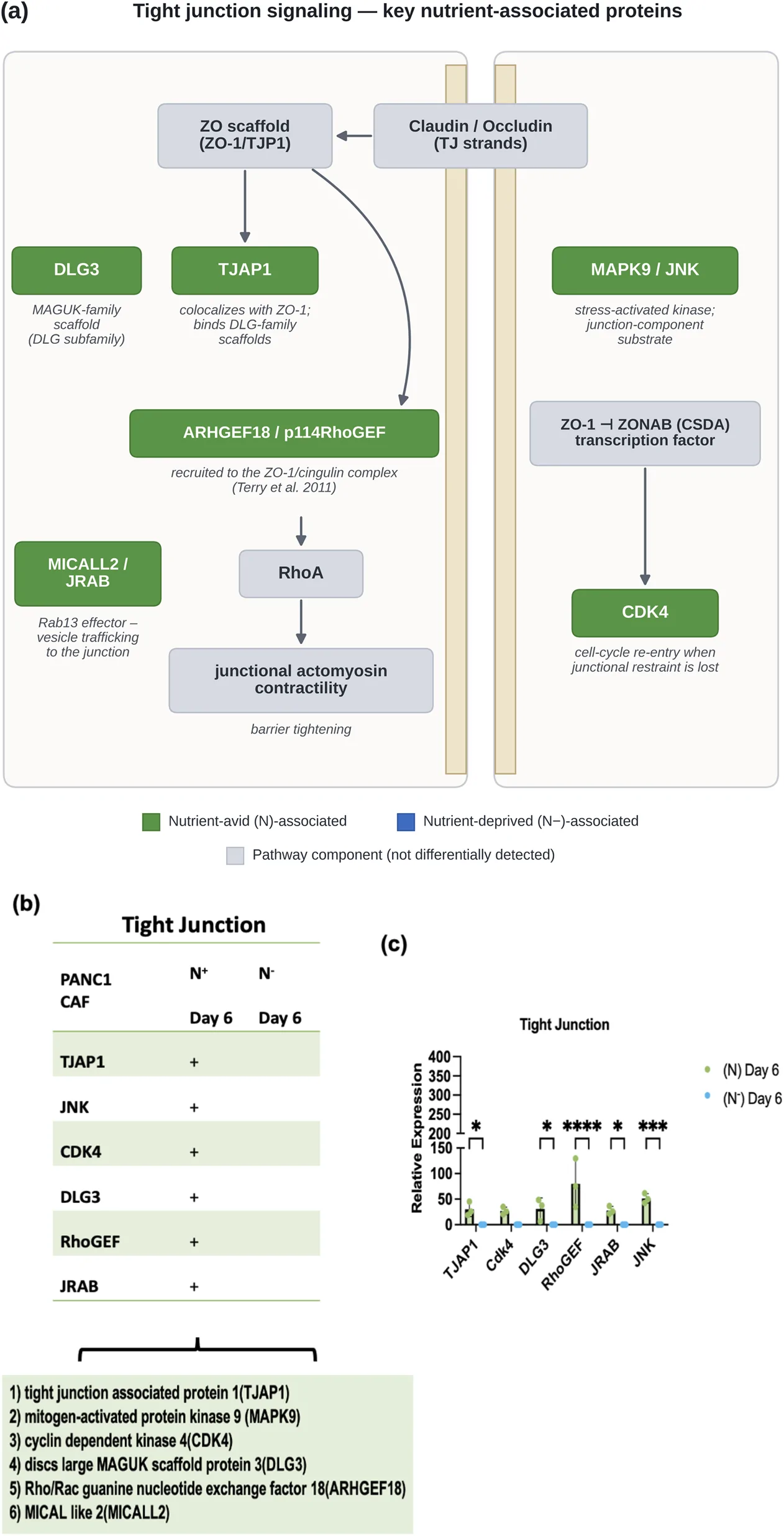
Alterations in tight junction-related proteins in response to nutrient availability. **(A)** Simplified schematic highlighting the principal nutrient-associated proteins identified in the proteomic analysis. Green indicates nutrient-avid (N)-associated proteins, blue indicates nutrient-deprived (N−)-associated proteins, and gray indicates pathway components included for context but not differentially detected. Original KEGG pathway maps are provided in Supplementary Figure S6 and S7. **(B)** Key nutrient-associated proteins detected at Day 6. **(C)** Relative protein abundance normalized to total protein concentration. Proteins detected in at least two of three biological replicates were considered reliably detected. Data are mean ± SD (n = 3). Statistical significance was determined by two-way ANOVA with Sidak’s multiple-comparisons test; p < 0.05, p < 0.01, p < 0.001.

**FIGURE 7:**
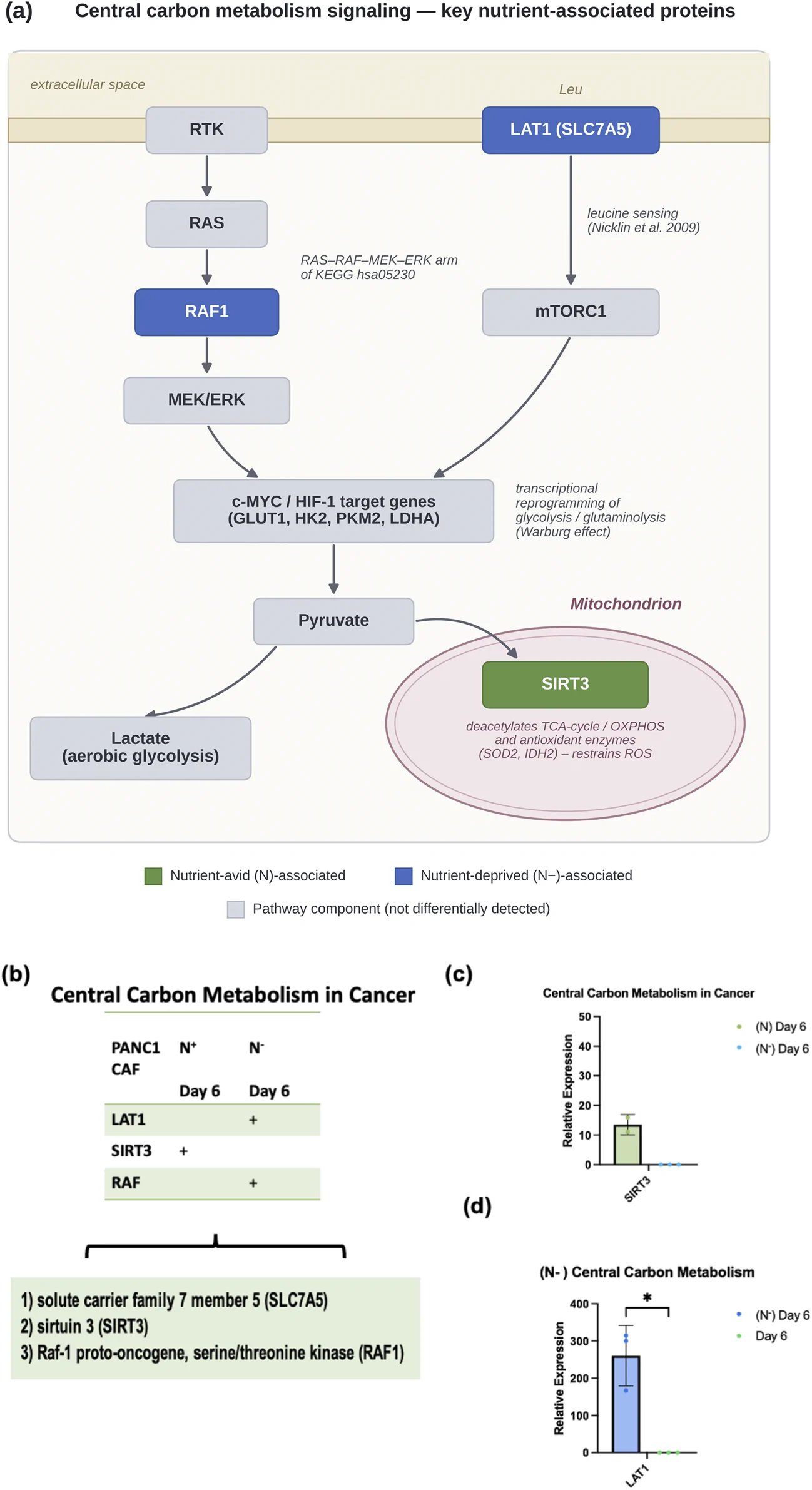
Alterations in central carbon metabolism-related proteins in response to nutrient availability. **(A)** Simplified schematic highlighting the principal nutrient-associated proteins identified in the proteomic analysis. Green indicates nutrient-avid (N)-associated proteins, blue indicates nutrient-deprived (N−)-associated proteins, and gray indicates pathway components included for context but not differentially detected. Original KEGG pathway maps are provided in Supplementary Figure S10 and S11. **(B)** Key nutrient-associated proteins detected at Day 6 **(C–D)** Relative protein abundance normalized to total protein concentration. Proteins detected in at least two of three biological replicates were considered reliably detected. Data are mean ± SD (n = 3). Statistical significance was determined using Welch’s t-test; p < 0.05, p < 0.01, p < 0.001.

### 2.5 Clinical relevance analysis using GEPIA

To assess the clinical relevance of the proteomic findings, a panel of 23 genes selected across the identified pathway signatures was evaluated using the Gene Expression Profiling Interactive Analysis (GEPIA) web tool. Transcript levels were compared between pancreatic adenocarcinoma tissues from The Cancer Genome Atlas (TCGA; n = 179) and normal pancreatic tissues from the Genotype-Tissue Expression (GTEx) project (n = 171). A p-value <0.05 was considered statistically significant. The results are presented in Figure 8.

**FIGURE 8:**
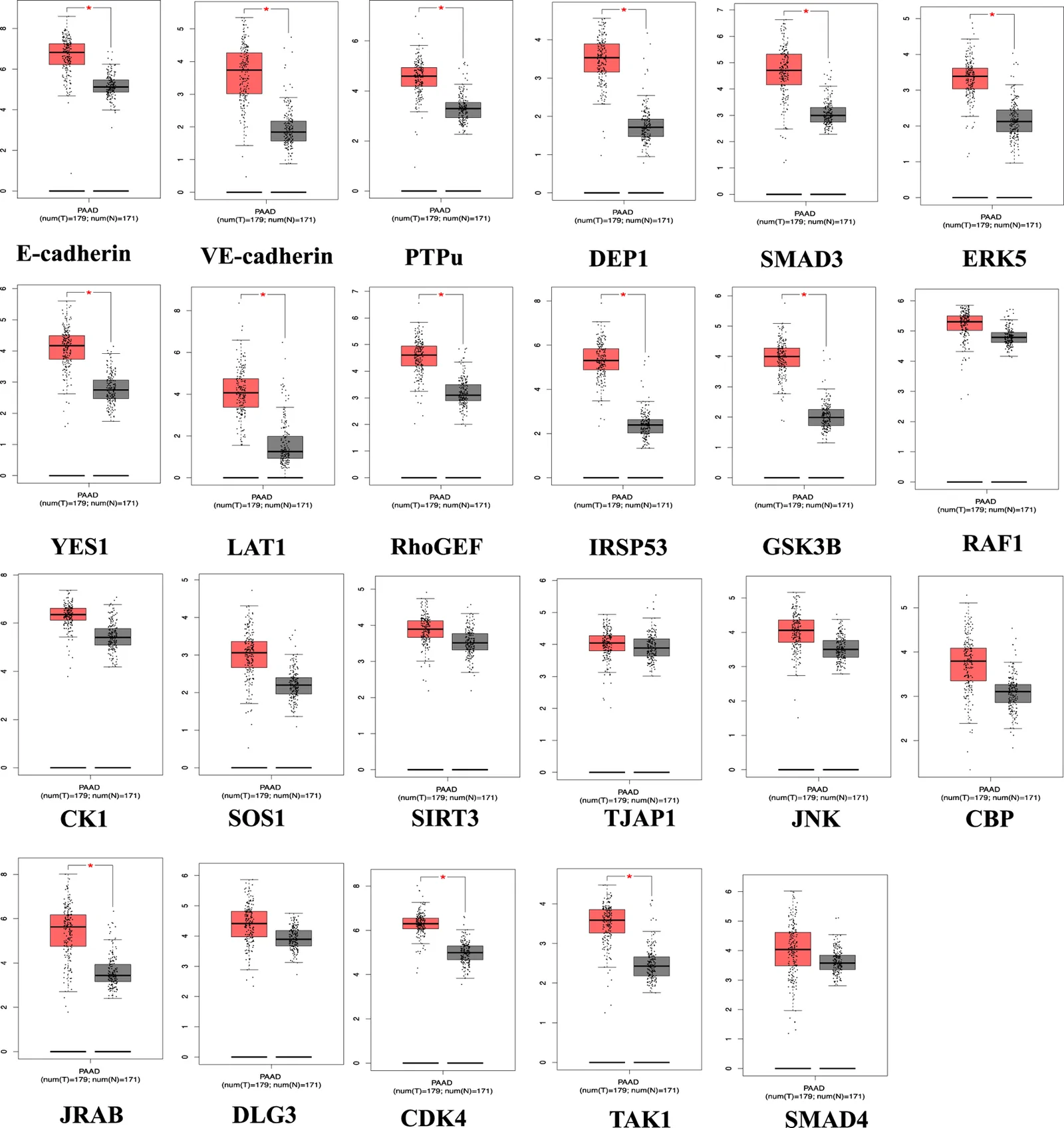
GEPIA analysis of gene expression in pancreatic adenocarcinoma and normal tissues. Boxplots show transcript levels of 23 selected genes in pancreatic adenocarcinoma (PAAD) tissues from TCGA (n = 179) and normal pancreatic tissues from GTEx (n = 171). Red boxes indicate PAAD samples and gray boxes indicate normal tissues. GEPIA, Gene Expression Profiling Interactive Analysis; PAAD, pancreatic adenocarcinoma. P < 0.05 was considered statistically significant.

### 2.6 Quantitative real-time PCR validation

Total RNA was isolated from 3D tumor constructs at defined experimental time points using a commercial RNA extraction kit according to the manufacturer’s protocol. RNA concentration and purity were assessed spectrophotometrically. Gene expression analysis was performed using probe-based TaqMan quantitative real-time PCR assays. Relative gene expression levels were normalized to the housekeeping gene GAPDH and calculated using the ΔΔCt method. Primer–probe assay identifiers are listed in Supplementary Table 2. Three independent biological replicates were analyzed per experimental condition.

### 2.7 Statistical analysis

Statistical analyses were performed using GraphPad Prism software. Statistical tests were selected according to the experimental design and variability of the proteomic datasets. Depending on the comparison, Welch’s t-test, or two-way ANOVA followed by Sidak’s multiple comparisons test was applied as appropriate. Benjamini–Hochberg multiple-testing correction was applied to pathway-enrichment analyses. Protein-level differential abundance comparisons were evaluated using the statistical tests specified above without an additional proteome-wide FDR correction. Accordingly, protein-level findings based on nominal p-values are interpreted as exploratory and hypothesis-generating rather than as FDR-controlled proteome-wide associations. Proteins identified in at least two of three biological replicates were considered reliably detected to minimize stochastic variability in proteomic detection. A p-value <0.05 was considered statistically significant.

## 3 Results

### 3.1 Structural response of bioprinted PDAC–CAF constructs to nutrient availability

The previously established sea-and-island pancreatic tumor construct (sandi PTC) was used to examine nutrient-associated molecular changes in a spatially organized PDAC–CAF microenvironment. In this model, RFP-labeled PDAC tumor islands are surrounded by a GFP-labeled CAF-containing stromal compartment within a three-dimensional bioprinted matrix (Figure 9). Representative fluorescence images demonstrated distinct temporal changes in the constructs cultured under nutrient-avid (N) and nutrient-deprived (N−) conditions. Under nutrient-avid conditions, the GFP-positive CAF-associated compartment remained readily detectable around the RFP-positive PANC-1 tumor islands. Under nutrient-deprived conditions, the CAF-associated GFP signal progressively decreased, particularly at the later time point, whereas RFP-positive tumor islands remained detectable.

**FIGURE 9:**
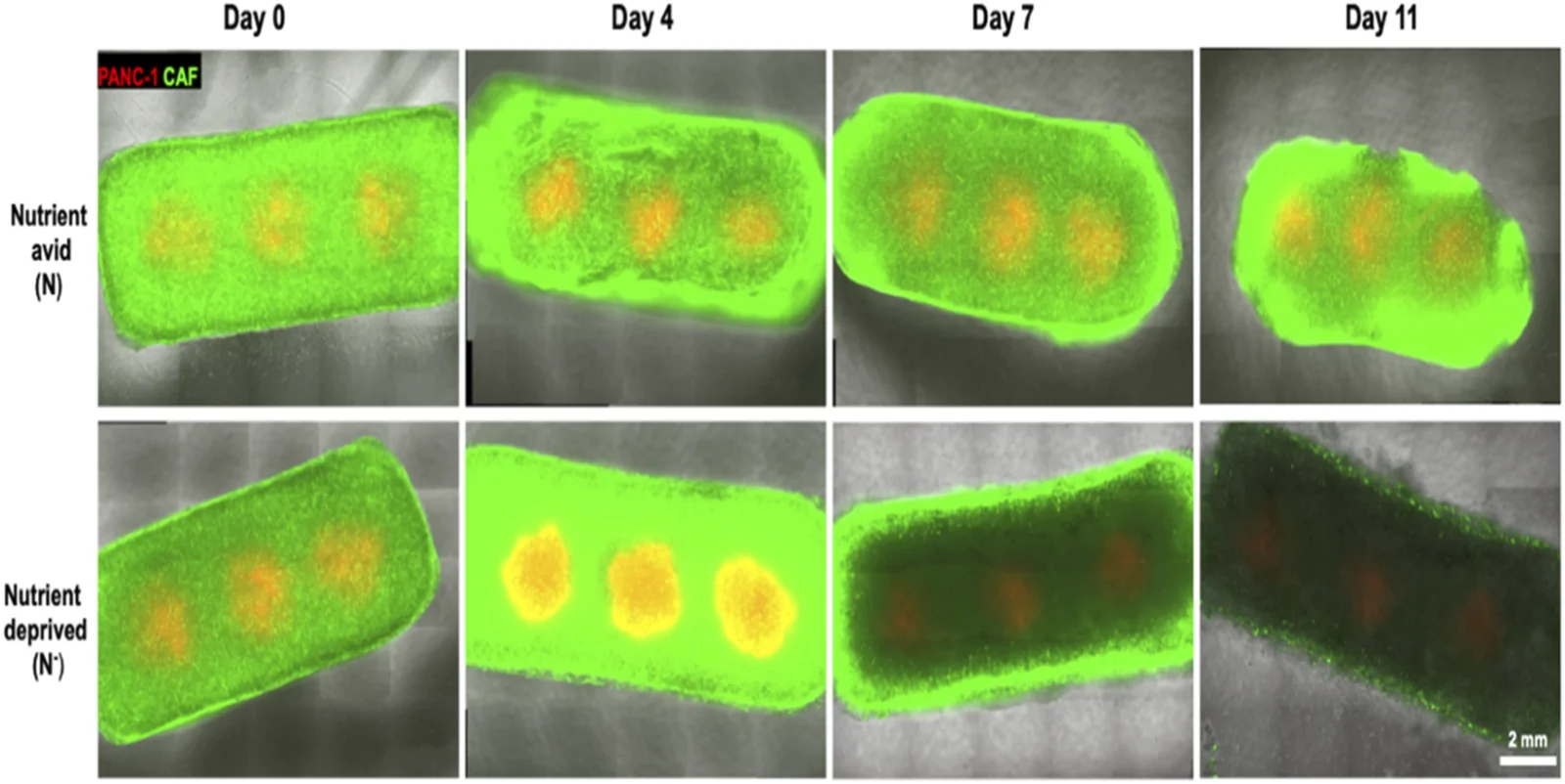
Alternation of the sea-island pancreatic tumor models cultured under different nutritional conditions. Bio printed sea-island pancreatic tumor constructs. PDAC islands (red) surrounded by dense CAFs (green), and were cultured within 3D-bioprinted collagen scaffolds under both nutrient-avid and nutrient-deprived conditions. The bioprinted tumor constructs were cultured in two distinct environments: nutrient-avid (N) and nutrient-deprived (N-). The cells were fluorescently labeled with GFP (for CAFs) and RFP (for PANC1) to visualize their distribution. The scale bar represents 2 mm. Corresponding images obtained from the CAPAN-2–CAF constructs are provided in Supplementary Figure S1.

The biological behavior of the sandi PTC under nutrient stress was characterized previously by Li et al. (Li et al., 2024). In that study, fluorescence-based measurements together with Ki67 and cleaved caspase-3 analyses were used to assess changes in PDAC and CAF abundance, proliferation, and apoptosis. Therefore, the fluorescence images presented in the current study are used to provide morphological context for the proteomic analysis and are not independently interpreted as direct measurements of CAF death or apoptosis.

Day 6 was selected as the principal time point for direct proteomic comparison between nutrient conditions. Previous characterization of the same model demonstrated that nutrient-dependent structural remodeling becomes evident during this intermediate period, whereas prolonged nutrient deprivation beyond approximately Day 7 is associated with progressive loss of model homeostasis and reduction of the CAF-associated compartment (Li et al., 2024). Because the proteomic measurements were obtained from bulk multicellular constructs, later-stage deterioration could increasingly introduce differences in cellular composition in addition to nutrient-dependent molecular regulation. Day 6 was therefore considered an appropriate intermediate stage for comparison of nutrient-associated proteomic signatures.

### 3.2 Nutrient-associated metabolic pathway signatures at day 6

Proteomic profiling revealed distinct metabolic pathway-enrichment patterns between nutrient-avid and nutrient-deprived PANC-1–CAF constructs at Day 6 (Figure 1).

Under nutrient-avid conditions, enriched metabolic pathways included pantothenate and CoA biosynthesis, β-alanine metabolism, arginine and proline metabolism, and selenocompound metabolism (Figure 1A). Proteins associated with these nutrient-avid pathway signatures included DPYS, DPYD, ALDH4A1, MTR, ALDH3B1/B2, and MLYCD (Figure 1C).

In contrast, nutrient-deprived constructs showed enrichment of pathways associated with biosynthesis of unsaturated fatty acids, fatty-acid elongation, riboflavin metabolism, folate-associated metabolism, antifolate resistance, and terpenoid backbone biosynthesis (Figure 1B). Proteins contributing to these pathway signatures included HACD2, ACP5, FLAD1, ABCC3, and TYMS (Figure 1D).

These findings demonstrate that nutrient availability was associated with differences in the representation of metabolic pathway-associated proteins within the bioprinted tumor constructs. Because metabolic flux was not directly measured, pathway enrichment was not interpreted as direct evidence of increased or decreased metabolic activity.

### 3.3 Nutrient-associated differences in gap junction-related proteins

Analysis of proteins assigned to gap junction signaling identified four proteins showing condition-associated detection patterns at Day 6: CK1, RAF1, SOS1, and ERK5 (Figure 2).

CK1 and RAF1 were preferentially detected under nutrient-deprived conditions, whereas SOS1 and ERK5 were preferentially detected under nutrient-avid conditions. Relative abundance measurements of these proteins are shown in Figures 2C,D.

These data indicate that nutrient availability was associated with differential representation of proteins linked to gap junction-related signaling. Functional changes in gap-junction communication were not directly measured in the present study.

### 3.4 Nutrient-associated differences in adherens junction-related proteins

Several proteins assigned to adherens junction signaling demonstrated nutrient-associated detection patterns at Day 6 (Figure 3).

E-cadherin (CDH1), PTPRM, VE-cadherin (CDH5), SMAD3, IRSp53, TAK1, and PTPRJ were preferentially detected under nutrient-avid conditions. In contrast, CBP and YES were preferentially detected under nutrient-deprived conditions.

Relative abundance measurements of the corresponding proteins are presented in Figures 3C,D. The observed differences identify nutrient-associated changes in proteins related to cell–cell junction organization and associated signaling networks. However, the present proteomic analysis does not directly measure junctional integrity, adhesion strength, or downstream pathway activity.

### 3.5 qPCR assessment of selected junction-associated proteomic findings

To independently assess selected candidates identified in the proteomic analysis, qPCR was performed for CDH1, CDH2, CDH5, PTPRJ, CBP, and SMAD4 under nutrient-avid and nutrient-deprived conditions (Figure 4).

CDH1 transcript abundance was higher under nutrient-avid conditions, consistent with the preferential proteomic detection of E-cadherin in the same condition. PTPRJ also showed a broadly concordant relationship between transcript- and protein-level measurements. CBP transcript abundance increased under nutrient-deprived conditions, consistent with its preferential proteomic detection under nutrient deprivation.

Not all targets demonstrated concordant transcript and protein patterns. CDH5 transcripts showed increased expression at Day 6 under nutrient-deprived conditions despite preferential detection of VE-cadherin protein under nutrient-avid conditions. Differences were also observed between SMAD-family transcript measurements and corresponding proteomic observations.

Overall, the qPCR results provided independent transcript-level support for selected proteomic findings while also demonstrating that transcript abundance and protein detection were not uniformly concordant.

### 3.6 Nutrient-associated differences in focal adhesion-related proteins

Analysis of proteins assigned to focal adhesion signaling identified GSK3β, SOS1, and RAF1 as proteins showing nutrient-associated detection patterns at Day 6 (Figure 5). GSK3β was preferentially detected under nutrient-deprived conditions, whereas SOS1 and RAF1 were also represented in other signaling pathways examined in this study. Relative abundance measurements are shown in Figure 5C. These observations indicate nutrient-associated differences in proteins linked to focal-adhesion-related signaling. Functional outcomes such as focal adhesion assembly, β-catenin activity, migration, or invasion were not directly evaluated.

### 3.7 Nutrient-associated differences in tight junction-related proteins

Six proteins assigned to tight junction-related signaling showed condition-associated detection patterns: TJAP1, CDK4, DLG3, ARHGEF18/RhoGEF, MICALL2/JRAB, and MAPK9/JNK (Figure 6).

All six proteins were preferentially detected under nutrient-avid conditions and were not reliably detected under nutrient-deprived conditions according to the predefined replicate criteria. Relative abundance measurements are presented in Figure 6C.

These findings indicate that nutrient availability was associated with differences in the representation of proteins involved in epithelial junction organization and polarity. The functional consequences of these proteomic differences were not directly assessed.

### 3.8 Nutrient-associated differences in central carbon metabolism-related proteins

Analysis of proteins assigned to central-carbon-metabolism-associated pathways identified LAT1/SLC7A5, SIRT3, and RAF1 as showing nutrient-associated patterns at Day 6 (Figure 7).

LAT1 and RAF1 were preferentially detected under nutrient-deprived conditions, whereas SIRT3 was preferentially detected under nutrient-avid conditions. Relative abundance measurements of LAT1 and SIRT3 are presented in Figures 7C,D.

These findings demonstrate nutrient-associated differences in proteins linked to amino-acid transport, mitochondrial regulation, and signaling pathways represented within central carbon metabolism. The current data do not directly establish alterations in amino-acid flux, mitochondrial respiration, HIF activity, or metabolic dependency (Table 1).

**TABLE 1:** Biological functions of proteins identified in the KEGG analysis. Annotations were derived from established databases, literature review, and bioinformatics analysis.

Genes/Proteins	Protein description	Functions of the protein	References	Expression (N vs. N^−^)
EP300; CREBBP CBP	Histone acetyltransferase p300; CREB-binding protein	Regulate gene activity, cell growth, and differentiation	​	↑ N-
Yes1	Tyrosine-protein kinase yes	Regulate cell growth, survival, and movement	23	↑ N-
LAT1	Solute carrier family 7 member 5 (SLC7A5)	Transport amino acids and other molecules into cells	24	↑ N-
RAF1; Araf; BRAF	RAF proto-oncogene serine/threonine-protein kinase; serine/threonine-protein kinase A-raf;serine/threonine-protein kinase B-raf	Regulate cell cycle, proliferation, and survival	22, 25	↑ N-
E-cadherin	Cadherin 1(CDH1)	Control cell movement and maturation	26	↑ N
PTPu	Protein tyrosine phosphatase receptor type M (PTPRM)	Control cell differentiation, proliferation, and movement	27	↑ N
DEP1	Protein tyrosine phosphatase receptor type J (PTPRJ)	Antiproliferative and tumor suppressor functions	28,29	↑ N
VE-cadherin	Cadherin 5 (CDH5)	Regulate cell processes and blood vessel development	30	↑ N
Smad3	SMAD member 3 (SMAD3)	Control cell growth, apoptosis, and metastasis	31	↑ N
IRSp53	BAR/IMD domain containing adapter protein 2 (BAIAP2)	Regulate actin polymerization and cell junction formation	​	↑ N
Smad4	SMAD family member 4 (SMAD4)	Suppress tumor growth and metastasis	32, 33	↑ N
TAK	TAK1 (MAP3K7)	Promote cell survival and regulate signaling pathways	34	↑ N
TJAP1	Tight junction associated protein 1 (TJAP1)	Assist in forming tight junctions between cells	35	↑ N
Cdk4	Cyclin dependent kinase 4 (Cdk4)	Regulate cell growth and division	36	↑ N
DLG3	Discs large MAGUK scaffold protein 3 (DLG3)	Maintain cell polarity and receptor organization	​	↑ N
RhoGEF	Rho/Rac guanine nucleotide exchange factor 18 (ARHGEF18)	Shape cell morphology and tight junctions	​	↑ N
JRAB	MICAL like 2 (MICALL2)	Control recycling of cell junction proteins and actin organization	37	↑ N
JNK	Mitogen-activated protein kinase 9 (MAPK9)	Regulate cell proliferation, differentiation, and transcription	38	↑ N
GSK-3β	Glycogen synthase kinase 3 beta (GSK3B)	Influence tumor development and resistance to chemotherapy	39	↑ N-
SIRT3	Sirtuin 3 (SIRT3)	Act as a tumor suppressor and regulate metabolism	40	↑ N
SOS1	SOS ras/Rac guanine nucleoitide exchange factor 1 (SOS1)	Regulate cell growth and division via RAS/MAPK pathway	41	↑ N
ERK5	Mitogen-activated protein kinase 7 (MAPK7)	Control cell proliferation and differentiation	42	↑ N
CK1	Casein kinase 1 delta (CSNK1D)	Regulate cellular stress, WNT pathways, and cell processes	21	↑ N-

### 3.9 Comparison with the CAPAN-2–caf model

Corresponding observations from CAPAN-2–CAF constructs showed partially overlapping nutrient-associated trends where comparable data were available. The use of PANC-1 and CAPAN-2 in the sandi PTC platform was previously established to represent PDAC models with distinct differentiation states and different interactions with the CAF/ECM compartment (Li et al., 2024). In the present study, the PANC-1–CAF model served as the primary system for the detailed pathway-focused proteomic comparison. CAPAN-2–CAF observations are therefore considered supportive rather than equivalent independent validation of the complete PANC-1 proteomic dataset, particularly where quantitative protein recovery was more variable.

## 4 Discussion

### 4.1 Nutrient availability is associated with distinct metabolic proteomic programs

The principal finding of this study is that nutrient availability was associated with distinct metabolic proteomic signatures in multicellular PDAC–CAF constructs. Nutrient-avid conditions were characterized by enrichment of pathways associated with pantothenate and CoA biosynthesis, β-alanine metabolism, and amino-acid metabolism, whereas nutrient deprivation was associated with greater representation of fatty-acid biosynthesis and elongation, riboflavin metabolism, folate-associated pathways, and terpenoid backbone biosynthesis. These patterns are consistent with the metabolic plasticity that characterizes PDAC (Pavlova et al., 2022; Suzuki et al., 2020; Słotwiński et al., 2021; Malvi et al., 2022). Rather than relying on a single metabolic program, pancreatic tumor cells can alter nutrient utilization according to environmental conditions and interactions with surrounding stromal populations (Sherman et al., 2017; Francescone et al., 2021; Pavlova et al., 2022; Suzuki et al., 2020; Słotwiński et al., 2021). The enrichment of CoA- and amino-acid-associated pathways under nutrient-avid conditions may therefore reflect a molecular environment compatible with biosynthetic activity when metabolic substrates are relatively abundant. In contrast, the representation of unsaturated-fatty-acid biosynthesis and fatty-acid-elongation pathways under nutrient deprivation is consistent with a shift toward a more lipid-associated molecular program (Auciello et al., 2019; Han et al., 2024; Daemen et al., 2015; Koundouros and Poulogiannis, 2020). This interpretation is conceptually consistent with previous reports describing distinct glycolytic and lipogenic metabolic states in PDAC and with studies demonstrating substantial metabolic plasticity in pancreatic cancer (Daemen et al., 2015; Karasinska et al., 2020; Malvi et al., 2022). However, the present study did not directly measure glucose utilization, fatty-acid synthesis, β-oxidation, oxygen consumption, extracellular acidification, or metabolic flux. Accordingly, the present data should not be interpreted as direct demonstration of a glycolytic-to-lipogenic metabolic switch.

Proteins such as HACD2, FLAD1, TYMS, and ABCC3 contributed to nutrient-deprived pathway signatures. Their detection identifies candidate components of the nutrient-associated response, but does not establish that these proteins are required for survival under nutrient deprivation. Functional perturbation studies will be necessary to distinguish proteins that actively mediate adaptation from those that are secondary markers of the altered cellular state.

### 4.2 Tumor–stromal interactions provide important context for the nutrient response

Interpretation of the present proteomic data must consider the multicellular nature of the sandi PTC. The CAF/ECM compartment is not simply a structural component of the model but has previously been shown to influence nutrient diffusion and PDAC survival (Sherman et al., 2017; Francescone et al., 2021; Auciello et al., 2019; Han et al., 2024). The original characterization of the sandi PTC demonstrated restricted molecular diffusion through CAF-containing regions and nutrient-dependent alterations in tumor and stromal cellular behavior (Li et al., 2024). Previous characterization also showed progressive changes in the CAF compartment during nutrient deprivation and demonstrated proliferative and apoptotic responses using Ki67 and cleaved caspase-3 staining (Li et al., 2024). Thus, the proteomic differences identified here occur within a dynamically changing tumor–stromal system rather than in isolated pancreatic cancer cells. Importantly, the CAF population itself can display substantial phenotypic plasticity (Öhlund et al., 2017; Elyada et al., 2019; Neuzillet et al., 2019). In the previously characterized sandi PTC system, nutrient-avid conditions were associated with α-SMA-high/IL-6-low CAF-associated patterns, whereas nutrient deprivation was associated with α-SMA-low/IL-6-high patterns consistent with a more inflammatory-like CAF state (Li et al., 2024). These observations provide relevant biological context for the current proteomic findings and highlight that nutrient-associated changes may arise from coordinated alterations in both tumor and stromal compartments. Nevertheless, the bulk proteomic approach used in the present study does not allow individual proteins to be definitively assigned to PANC-1 cells or CAFs. The nutrient-associated signatures may therefore reflect changes in tumor cells, stromal fibroblasts, or both. Spatial proteomics, single-cell analyses, or cell-type-specific validation will be required to resolve the cellular origin of these molecular changes.

### 4.3 CAF heterogeneity adds an additional layer of complexity

CAF heterogeneity is particularly relevant when interpreting stromal responses in PDAC (Öhlund et al., 2017; Neuzillet et al., 2019). Multiple CAF states have been identified in pancreatic cancer, including myofibroblastic and inflammatory fibroblast populations, and their relative abundance and functional behavior can vary according to spatial location and microenvironmental conditions (Öhlund et al., 2017; Neuzillet et al., 2019).

The previous characterization of the sandi PTC showed that nutrient availability was associated with changes in α-SMA and IL-6 expression consistent with dynamic CAF phenotypic remodeling (Li et al., 2024). These results suggest that the nutrient environment itself may contribute to changes in the functional state of the fibroblast compartment (Sherman et al., 2017; Francescone et al., 2021; Auciello et al., 2019; Han et al., 2024; Li et al., 2024).

However, the present study was not designed to perform comprehensive CAF subtype classification. Thus, individual proteomic changes identified here cannot be specifically assigned to myCAF, iCAF, or other CAF populations (Li et al., 2024). Furthermore, the sandi PTC contains tumor cells, CAFs, and ECM but does not reproduce the complete cellular complexity of human PDAC, including immune, endothelial, and other stromal populations. These factors should be considered when extrapolating the findings to the human tumor microenvironment (Sarantis et al., 2020; Sherman and Beatty, 2023; Truong and Pauklin, 2021).

The role of lipid handling within CAFs also warrants particular attention. Lipid droplets and lipid-associated metabolic programs have been implicated in fibroblast activation and tumor–stromal metabolic interactions (Auciello et al., 2019; Han et al., 2024); however, lipid-droplet accumulation was not directly assessed in the present study. Therefore, the enrichment of lipid-associated pathways under nutrient deprivation cannot be used to infer CAF lipid-droplet formation.

### 4.4 Nutrient availability is associated with remodeling of junction-related protein networks

In addition to metabolic pathways, the proteomic dataset identified differences in proteins assigned to gap-junction, adherens-junction, focal-adhesion, and tight-junction signaling.

Under nutrient-avid conditions, proteins including E-cadherin, VE-cadherin, PTPRM, PTPRJ, SOS1, ERK5, TJAP1, DLG3, ARHGEF18, MICALL2, JNK, and SIRT3 were preferentially represented. In contrast, CK1, RAF1, YES, CBP, GSK3β, and LAT1 were preferentially associated with nutrient-deprived conditions.

The preferential detection of E-cadherin and related junction-associated proteins under nutrient-avid conditions is consistent with a cellular state in which epithelial and intercellular organization is more strongly represented (Petrova et al., 2016; Vestweber, 2008; Feigin and Muthuswamy, 2009). Previous characterization of the same sandi PTC platform also reported higher E-cadherin expression under nutrient-avid conditions in both PANC-1 and CAPAN-2 constructs (Li et al., 2024). In contrast, reduced representation of selected adhesion-associated proteins during nutrient deprivation may be compatible with structural remodeling of the multicellular construct.

However, these proteomic changes should not be equated directly with functional loss of junctional integrity or induction of epithelial–mesenchymal transition. The present study did not directly measure junctional localization, epithelial permeability, cell migration, invasion, or EMT state. Accordingly, the results identify nutrient-associated remodeling of junction-related protein networks rather than demonstrating a specific functional transition.

### 4.5 Selected qPCR findings provide partial independent support

The qPCR analysis provided an independent transcript-level assessment of selected candidates identified in the proteomic analysis. Concordant patterns for CDH1, PTPRJ, and CBP support the presence of nutrient-associated molecular differences detected by the proteomic screen.

However, the relationship between transcript and protein abundance was not uniform. For example, CDH5 showed a transcript-level pattern that differed from its proteomic detection pattern. Such discordance is not unexpected because mRNA abundance is influenced by transcriptional regulation, whereas protein abundance additionally reflects translational efficiency, protein turnover, degradation, and post-translational regulation.

Therefore, the qPCR experiments should be interpreted as partial independent support for selected molecular observations rather than as complete validation of the proteomic dataset or proof of pathway activity. Additional protein-level validation would strengthen these observations but was not performed in the current study.

### 4.6 PANC-1 and CAPAN-2 highlight the importance of PDAC heterogeneity

PDAC is molecularly heterogeneous, and this heterogeneity can influence tumor–stromal interactions and metabolic responses (Daemen et al., 2015; Karasinska et al., 2020; Puleo et al., 2018). The sandi PTC platform was previously characterized using both PANC-1 and CAPAN-2 cells, which represent distinct differentiation states. Previous work demonstrated differences in construct morphology, CAF behavior, and ECM remodeling between these models (Li et al., 2024).

In the present study, the PANC-1–CAF system was used for the primary detailed proteomic analysis, while CAPAN-2–CAF observations provided supporting evidence of partially overlapping nutrient-associated responses. This comparison suggests that at least some nutrient-responsive signatures may not be unique to a single PDAC cell line.

Nevertheless, the CAPAN-2 data were not quantitatively equivalent for every analysis, and the use of two established cell lines cannot capture the full molecular diversity of human PDAC. The findings should therefore not be generalized to all PDAC tumors. Future studies incorporating additional cell lines, patient-derived organoids, primary tumor cells, or patient-derived CAF combinations will be important for determining the broader reproducibility of these nutrient-associated signatures (Boj et al., 2015; Driehuis et al., 2019).

### 4.7 Biological and translational implications

Several proteins identified in the present study, including CK1, RAF1, GSK3β, LAT1, ERK5 are involved in signaling or metabolic processes relevant to cancer biology (Knippschild et al., 2014; Mollinedo and Gajate, 2019; Zhang et al., 2020; Collisson et al., 2012; Ding and Billadeau, 2020; Hoang et al., 2017). Additional nutrient-associated candidates identified here, including TYMS, FLAD1, and HACD2, warrant further investigation.

However, differential protein representation alone is insufficient to establish therapeutic relevance. The current data do not demonstrate that pharmacological or genetic targeting of these proteins alters PDAC survival, metabolism, or tumor–stromal interactions. Accordingly, these proteins should be considered candidate molecular vulnerabilities rather than validated therapeutic targets.

The previously published characterization of the sandi PTC demonstrated that proteomic signatures generated using this platform can be compared with human PDAC datasets and patient-survival information, supporting the potential translational relevance of the model (Li et al., 2024). Nevertheless, the specific candidate proteins identified in the present pathway-focused analysis require dedicated validation in independent patient datasets before conclusions regarding biomarker or prognostic significance can be made.

Future integration of the present candidates with human PDAC transcriptomic and proteomic datasets, including TCGA, CPTAC, or comparable patient cohorts, may help prioritize those proteins that show both nutrient-associated regulation in the experimental model and clinical relevance in human disease.

### 4.8 Study scope and mechanistic limitations

The present study was designed primarily as a systems-level proteomic investigation of nutrient-associated molecular patterns rather than as a functional perturbation study. Consequently, pathway enrichment and differential protein detection provide evidence of association but do not establish causality. Pharmacological inhibition or genetic manipulation of representative candidates such as TYMS, FLAD1, HACD2, CK1, ERK5, or LAT1 would be required to determine whether these proteins actively contribute to survival or adaptation under nutrient deprivation. Such experiments were not performed in the present study. Similarly, the absence of direct measurements of metabolic flux, mitochondrial respiration, extracellular acidification, lipid accumulation, junctional function, migration, or invasion limits mechanistic interpretation. The present findings should therefore be considered hypothesis-generating and provide a framework for future targeted studies rather than definitive evidence of specific adaptive mechanisms. A further limitation is the focus of the principal direct proteomic comparison on Day 6. This time point was selected on the basis of the previously characterized temporal behavior of the model, which demonstrated increasing loss of homeostasis under prolonged nutrient deprivation beyond approximately Day 7 (Li et al., 2024). Nevertheless, longitudinal proteomic profiling across additional intermediate time points would provide a more comprehensive understanding of the temporal evolution of the nutrient response.

## 5 Conclusion

This study identified distinct nutrient-associated metabolic and junction-related proteomic signatures in 3D bioprinted PDAC–CAF tumor constructs. Nutrient-avid and nutrient-deprived conditions were associated with different representations of proteins involved in metabolic pathways, cell–cell interactions, cell–matrix signaling, and stress-associated processes. Selected proteomic observations were supported at the transcript level by qPCR, although transcript and protein measurements were not uniformly concordant. The findings therefore provide systems-level, hypothesis-generating evidence of nutrient-associated molecular remodeling rather than direct demonstration of causal metabolic or signaling mechanisms. Together with the previously established biological characterization of the sandi PTC platform, these results support the use of the model for investigating tumor–stromal responses to nutrient availability. Functional perturbation studies and validation in independent PDAC models and patient datasets will be required to establish the mechanistic and translational significance of the candidate proteins identified in this study.

## Data Availability

The original contributions presented in the study are included in the article/Supplementary Material, further inquiries can be directed to the corresponding author.
