# Supplementary material for: Proteomic analysis of bioprinted pancreatic tumor models under different nutrient conditions
Source: Front Bioeng Biotechnol. 2026 Oct 5;14:1921652. doi: 10.3389/fbioe.2026.1921652 (PMC13678492; doi:10.3389/fbioe.2026.1921652)
Supplement: Supplementary file 1 [file Supplementaryfile1.docx]

**Supplementary Materials and Methods**
Proteomic Analysis of Bioprinted Pancreatic Tumor Models Under Different Nutrient Conditions

**Supplementary Figure S1. Alternation of the sea-island pancreatic tumor. Capan-2&CAF organoids cultured under different nutritional conditions**. Organoids were cultured in two distinct conditions nutrient-avid (N) and nutrient-deprived (N-). The cells were fluorescently labeled with GFP (for CAF) and RFP (for Capan2) to visualize their distribution. The scale bar represents 2 mm. Work conducted by Ming Li at the Ye Lab.

**Supplementary Figure S2. Adherens Junctions Alternation in Panc1- CAF Organoids under Different Nutrient Exposures.** KEGG pathway analysis was performed to investigate the alterations in adherens junction signaling pathways subjected to varying nutrient conditions at day 6. (N-): nutrition deprived.

**Panc1 Day 6**

**Panc1**

**Day 6**

**N**

**(**

**-**

**6**

**Panc1 Day**

**)**

**Supplementary Figure S3. Adherens Junctions Alternation in Capan2-CAF Organoids under Different Nutrient Exposures.** KEGG pathway analysis was performed to investigate the alterations in adherens junction signaling pathways subjected to varying nutrient conditions at day 6. (N-): nutrition deprived.

**Capan2**

**-**

**Day 6**

**N**

**(**

**-**

**6**

**)**

**Capan2 Day**

**Supplementary Figure S4. Gap Junctions Alternation in Panc1-CAF Organoids under Different Nutrient Exposures.** KEGG pathway analysis was performed to investigate the alterations in gap junction signaling pathways subjected to varying nutrient conditions at day 6.

(N-): nutrition deprived.

**Panc1 Day 6**

**(**

**N**

**-**

**)**

**Panc1 Day**

**6**

**Supplementary Figure S5. Gap Junctions Alternation in Capan2-CAF Organoids under Different Nutrient Exposures.** KEGG pathway analysis was performed to investigate the alterations in gap junction signaling pathways subjected to varying nutrient conditions at day 6.

(N-): nutrition deprived.

**Capan2 Day 6**

**(N-)**

**Capan2 Day 6**

**Figure 5. Tight Junctions Alternation in P**

**anc**

**1**

**-**

**CAF Organoids under Different Nutrient**

**Exposures.**

KEGG pathway analysis was performed to

investigate the alterations in tight junction

signaling pathways in Panc1

-

CAF organoids subject

varying nutrient conditions at day

6.

(

N

-

):

nutriti

o

n deprive

d

**Supplementary Figure S6. Tight Junctions Alternation in Panc1-CAF Organoids under Different Nutrient Exposures.** KEGG pathway analysis was performed to investigate the alterations in tight junction signaling pathways subjected to varying nutrient conditions at day 6. (N-): nutrition deprived.

**Panc1 Day 6**

**(**

**N**

**-**

**)**

**Panc1 Day**

**6**

**Supplementary Figure S7. Tight Junctions Alternation in Capan2-CAF Organoids under Different Nutrient Exposures.** KEGG pathway analysis was performed to investigate the alterations in tight junction signaling pathways subjected to varying nutrient conditions at day 6. (N-): nutrition deprived.

**Capan2 Day 6**

**(**

**N**

**-**

**)**

**Capan2 Day**

**6**

**Supplementary Figure S8. Focal Adhesion Alternation in Panc1-CAF Organoids under Different Nutrient Exposures.** KEGG pathway analysis was performed to investigate the alterations in focal junction signaling pathways subjected to varying nutrient conditions at day 6. (N-): nutrition deprived.

**Panc1 Day 6**

### (N-) Panc1 Day 6

**Supplementary Figure S9. Focal Adhesion Alternation in Capan2-CAF Organoids under Different Nutrient Exposures.** KEGG pathway analysis was performed to investigate the alterations in focal junction signaling pathways subjected to varying nutrient conditions at day 6. (N-): nutrition deprived.

**Capan2 Day 6**

**(**

**N**

**-**

**)**

**Capan2 Day**

**6**

**Supplementary Figure S10. Central Carbon Metabolism in Cancer Alternation in Panc1-CAF Organoids under Different Nutrient Exposures.** KEGG pathway analysis was performed to investigate the alterations in central carbon metabolism in cancer signaling pathways in Panc1-CAF organoids subjected to varying nutrient conditions at day 6. (N-): nutrition deprived.

**Panc1 Day 6**

**(**

**N**

**-**

**)**

**Panc1 Day**

**6**

**Supplementary Figure S11. Central Carbon Metabolism in Cancer Alternation in Capan2-CAF Organoids under Different Nutrient Exposures.** KEGG pathway analysis was performed to investigate the alterations in central carbon metabolism in cancer junction signaling pathways in Capan2-CAF organoids subjected to varying nutrient conditions at day 6. (N-): nutrition deprived.

**Capan2 Day 6**

### (N-) Capan2 Day 6

**Supplementary Figure S12. STRING network of the differentially expressed proteins (DEPs) identified in nutrient rich and nutrient-scarce conditions.** STRING analysis can help uncover interactions and relationships among proteins that differ in nutrient-rich and nutrient-deprived environments. By mapping these interactions, STRING provides insights into how these proteins work together within specific biological pathways or processes. This can reveal critical connections, identify key regulatory proteins, and suggest new hypotheses about how nutrient conditions affect cellular behavior. Ultimately, it can guide further research by highlighting potential therapeutic targets and helping to prioritize which proteins or pathways to focus on in future experiments.

**Supplementary Figure S13.** Venn diagram showing the distribution of expressed proteins in Panc1 and Capan2 organoids under nutrient-sufficient and nutrient-deprived conditions on Day 6. (N-): Nutrition-deprived.

**Supplementary Figure S14. Gene Ontology (GO) Enrichment Analysis**. GO enrichment analysis was conducted based on molecular function, biological process, and cellular component categories. The top 10 GO terms were identified for Day 6 and (N-) Day 6. n = 3 for each group. Data are presented as mean ± standard error of the mean (SEM). Proteins that were expressed at least twice were included in the day-by-day GO analysis. (N-): Nutrition-deprived.

**Supplementary Figure S15. Gene Ontology (GO) Enrichment Analysis**.

## Statistical results for section 2.1

| **Signaling Pathways exclusively activated at Day 6** | **p-value** |
| --- | --- |
| Pantothenate and CoA biosynthesis | 6.10E-02 |
| Tuberculosis | 3.20E-02 |
| Chagas disease | 7.60E-03 |
| beta-Alanine metabolism | 2.30E-04 |
| Arginine and proline metabolism | 7.30E-02 |
| Relaxin signaling pathway | 9.00E-03 |
| Cell cycle | 5.50E-02 |
| Viral myocarditis | 6.40E-02 |
| Human T-cell leukemia virus 1 infection | 2.00E-02 |
| Endocrine resistance | 4.80E-02 |
| Apoptosis - multiple species | 6.10E-02 |
| Toxoplasmosis | 1.40E-03 |
| Longevity regulating pathway - multiple species | 3.60E-02 |
| Estrogen signaling pathway | 1.30E-02 |
| Measles | 3.40E-03 |
| Seleno compound metabolism | 9.60E-02 |
| Nucleotide excision repair | 8.90E-02 |
| Mismatch repair | 4.90E-02 |
| Endocrine and other factor-regulated calcium reabsorption | 4.10E-02 |
| Hypertrophic cardiomyopathy | 9.60E-02 |
| Antigen processing and presentation | 8.80E-02 |
| Rheumatoid arthritis | 8.50E-02 |
| Colorectal cancer | 2.90E-02 |
| ATP-dependent chromatin remodeling | 7.90E-02 |
| Alcoholic liver disease | 2.72E-02 |
| Arrhythmogenic right ventricular cardiomyopathy | 8.70E-02 |
| T cell receptor signaling pathway | 6.10E-02 |

| **Signaling Pathways exclusively activated at (N-) Day 6** | **p-value** |
| --- | --- |
| Biosynthesis of unsaturated fatty acids | 1.50E-02 |
| Prostate cancer | 7.60E-02 |
| Chemokine signaling pathway | 8.00E-02 |
| Riboflavin metabolism | 3.80E-02 |
| Folate biosynthesis | 8.60E-02 |
| Thyroid cancer | 1.00E-02 |
| Antifolate resistance | 3.90E-02 |
| Terpenoid backbone biosynthesis | 6.60E-02 |
| Fatty acid elongation | 5.20E-03 |

**(b) (a)**

**(c)**

**Supplementary Figure S16**. Panc1 & CAF enrichened pathways p-values on Day 6 under (b) nutrition-avid (c) or –deprived conditions.

**(a)**

**(b)**

**Supplementary Figure S17.** Panc1 & CAF enrichened pathways on Day 6 under (a) nutrition-avid (b) or –deprived conditions.

| **Signaling Pathways exclusively activated at Day 6** | **p-value** |
| --- | --- |
| Pantothenate and CoA biosynthesis | 5.70E-02 |
| beta-Alanine metabolism | 2.00E-04 |
| Arginine and proline metabolism | 6.60E-02 |
| Cell cycle | 7.10E-02 |
| Viral myocarditis | 7.90E-02 |
| Human T-cell leukemia virus 1 infection | 3.30E-02 |
| Endocrine resistance | 3.70E-02 |
| Apoptosis - multiple species | 5.60E-02 |
| Longevity regulating pathway - multiple species | 6.70E-02 |
| Estrogen signaling pathway | 2.10E-02 |
| Selenocompound metabolism | 9.10E-02 |
| Nucleotide excision repair | 4.70E-02 |
| Mismatch repair | 4.50E-02 |
| Endocrine and other factor-regulated calcium reabsorption | 3.70E-02 |
| Hypertrophic cardiomyopathy | 7.30E-02 |
| Rheumatoid arthritis | 8.50E-02 |
| ATP-dependent chromatin remodeling | 6.80E-02 |
| Alcoholic liver disease | 8.50E-02 |
| Arrhythmogenic right ventricular cardiomyopathy | 6.60E-02 |
| T cell receptor signaling pathway | 8.50E-02 |

| **Signaling Pathways exclusively activated at (N-) Day 6** | **p-value** |
| --- | --- |
| Biosynthesis of unsaturated fatty acids | 1.80E-02 |
| Chemokine signaling pathway | 6.90E-02 |
| PPAR signaling pathway | 8.00E-02 |
| Riboflavin metabolism | 4.10E-02 |
| Folate biosynthesis | 9.80E-02 |
| Neurotrophin signaling pathway | 6.70E-02 |
| Antifolate resistance | 4.60E-02 |
| Antigen processing and presentation | 8.90E-02 |
| Terpenoid backbone biosynthesis | 7.50E-02 |

**(a)	(b)**

**(c)**

**Supplementary Figure S18.** Capan2 & CAF enrichened pathways p-values on Day 6 under (b) nutritionavid or (c) – deprived conditions.

**(a)**

**(b)**

**Supplementary Figure S19.** Capan2 & CAF enrichened pathways on Day 6 under (a) nutrition-avid or (b) – deprived conditions.

**Supplementary Table 2.** Primers and probes for qRT-PCR

| **Gene** | **Assay ID (Applied Biosystems)** |
| --- | --- |
| CDH1 | Hs01023894_m1 |
| CDH2 | Hs00983056_m1 |
| CDH5 | Hs00901465_m1 |
| SMAD4 | Hs00929647_m1 |
| PTPRJ | Hs01119326_m1 |
| CREBBP | Hs00932878_m1 |
| GAPDH | Hs02786624_g1 |
